# Outcomes of endodontic microsurgery using different calcium silicate–based retrograde filling materials: a cohort retrospective cone-beam computed tomographic analysis

**DOI:** 10.1186/s12903-023-02782-w

**Published:** 2023-02-03

**Authors:** Rawan F. Eskandar, Mey A. Al-Habib, Mohammed A. Barayan, Hadeel Y. Edrees

**Affiliations:** 1 Department of Endodontics, Faculty of Dentistry, King Abdulaziz University, P.O. Box 80209, Jeddah, 21589 Saudi Arabia; 2 Oral Basic Science, Oral and Maxillofacial Radiology Department, Faculty of Dentistry, King Abdulaziz University, P.O. Box 80209, Jeddah, 21589 Saudi Arabia

**Keywords:** Endodontic microsurgery, Calcium silicate–based material, Lid technique, Cone-beam computed tomography, Volumetric analysis

## Abstract

**Purpose:**

To evaluate the outcomes of endodontic microsurgery (EMS) using mineral trioxide aggregate (MTA; Dentsply Sirona, Charlotte, NC, USA), EndoSequence root repair material (RRM putty; Brasseler, Savannah, GA), and injectable Bioceramic (BC) sealer (Brasseler USA) followed by the application of RRM putty (lid technique) as root-end filling materials.

**Methods:**

One hundred and ten patients who underwent EMS between 2016 and 2020 at King Abdulaziz University Dental Hospital were recruited for clinical and radiographic follow-up after a minimum of 1 year. Radiographic assessment was performed using periapical radiographs (PAs) and cone-beam computed tomography (CBCT). Volumetric analysis of periapical radiolucencies (PARLs) was performed using Amira software.

**Results:**

Seventy-nine patients (103 teeth: MTA group, n = 28; RRM putty, n = 41; lid technique, n = 34), attended the follow-up visit, with an average follow-up period of 24 months (recall rate = 74.5%). Of the 103 teeth, 40 were anteriors, 24 were premolars, and 39 were molars. All three groups of retrograde filling materials (MTA, RRM putty, and lid technique) showed high success rates on both PA (85.7, 85.4, 94.1%, respectively) and CBCT imaging (67.9, 75.6, 88.2%, respectively), without any significant difference among the success rates of different materials. Overall, a slight agreement was noted between the PA and CBCT outcomes, with a statistically significant difference (*P* = 0.029). None of the patient-, tooth-, or treatment-related factors significantly influenced the outcomes of EMS. Adequate density of root canal filling material was significantly associated with a high percentage of completely healed cases on CBCT (*P* = 0.044). PARL volumes were reduced significantly over 1–4 years follow-up after EMS (*P* < 0.001)

**Conclusions:**

EMS showed high success rates on both PA and CBCT when MTA, RRM putty or lid technique were used as retrograde filling materials. CBCT imaging is more precise than PA in detecting the healing outcomes of EMS.

## Background

Endodontic surgery (ES) is a reliable treatment option for the preservation of teeth with post-treatment apical periodontitis after the failure of non-surgical approaches [1]. Modern microsurgical techniques have been characterized using an operating microscope, ultrasonic tips, and biocompatible retrograde filling materials such as calcium silicate–based materials [2]. Modern techniques have significantly improved the success rate of ES compared with that of traditional techniques [3].

Mineral trioxide aggregate (MTA) (ProRoot MTA; Dentsply, Tulsa, OK, USA) was the first calcium silicate–based retrograde filling material and is characterized by its biocompatibility, excellent sealing ability, antibacterial properties, and regenerative capabilities [4–6]. Other calcium silicate–based materials, such as EndoSequence Root Repair Material (RRM putty; Brasseler, Savanna, GA, USA), were subsequently introduced. RRM is a premixed moldable putty that is similar to MTA but has additional advantages such as superior handling properties and washout resistance [7]. RRM putty showed significantly greater healing on the root-end surface and periapical area than MTA in a dog model; this superiority in healing was also observed in cone-beam computed tomography (CBCT) and micro–computed tomography images but not in periapical radiographs (PAs) [8]. MTA and RRM putty as retrograde filling materials were also evaluated in a randomized controlled trial by Safi et al. [9], who assessed the outcomes of endodontic microsurgery (EMS) using PA and CBCT imaging. The two materials exhibited high success rates of EMS in both imaging modalities, with no statistically significant difference.

Recently, the Buffalo study added a new group to the comparison alongside MTA and RRM putty; this new group was treated by the lid technique (using injectable bioceramic [BC] sealer (Brasseler, Savanna, GA, USA) followed by application of RRM putty). The combined clinical and CBCT outcomes indicated high success rates for all three groups of retrograde filling materials, with no statistically significant differences [10]. In the last few years, the use of the lid technique for retrograde filling has increased; however, limited data are available regarding the outcomes of this technique.

The use of CBCT for the assessment of EMS outcomes has increased considerably because it has higher sensitivity and specificity than PAs in identifying post-treatment apical periodontitis [11]. Additionally, CBCT enables the determination of volumetric changes in periapical radiolucencies (PARLs) after EMS, representing the objective outcomes. However, only a few studies have assessed the outcome of EMS and determined the prognostic factors that may affect the outcomes by CBCT. Cases of guided tissue regeneration (GTR) and bone graft were included in the analyses of these studies [9, 10], which may have prevented accurate radiographic evaluation. Moreover, volumetric changes in PARLs after EMS have been investigated in a limited number of studies with small sample sizes [11, 12].

Thus, the aims of this retrospective study were to (1) evaluate the clinical and radiographic outcomes of MTA, RRM putty, and the lid technique as a retrograde filling material using 2-dimensional (2D) PAs and 3-dimensional (3D) CBCT imaging; (2) identify the potential prognostic factors of first-time EMS; (3) compare the outcomes of EMS using 2D versus 3D imaging; and (4) evaluate the volumetric changes in PARLs after EMS.

## Methods

### Study design and case selection

Participants eligible for follow-up were identified retrospectively from a clinical database including patients who had received EMS from postgraduate endodontic residents and faculty at the Department of Endodontics of KAU Dental Hospital (KAUDH) between October 2016 and February 2020. A total of 110 patients (142 teeth) were eligible for follow-up according to the inclusion and exclusion criteria listed below.

The inclusion criteria were as follows:

1. American Society of Anesthesiologists (ASA) class I or II
2. Teeth with adequate coronal coverage
3. Use of calcium silicate–based retrograde filling material (MTA, RRM putty, or BC sealer followed by RRM putty)
4. Follow-up period of at least 1 year
5. Cases with complete pre- and postoperative radiographs and clinical notes

The exclusion criteria were as follows:

1. Cases of GTR, bone graft, and re-surgery
2. Teeth with through-and-through defects
3. Teeth with microsurgical classification D, E, or F defined by Kim and Kratchman [2]
4. Extraction of teeth after EMS for non-endodontic reasons
5. Teeth with vertical root fracture detected during surgery
6. Cases combined with perforation or resorptive defect
7. Pregnant patients

### Preoperative examination and treatment protocol

Prior to EMS, the patients underwent detailed clinical and radiographic examination, which included PA using a KaVo X-ray machine (KaVo FOCUS, Tuusula, Finland) and CBCT using one of the following machines available in the dental hospital at the time of surgery:

1. Between October 2016 and November 2020, an i-CAT scanner (Imaging International Sciences, Hatfield, Pennsylvania, USA) with a field of view (FOV) = 8 cm × 8 cm, and a voxel size of 0.125 mm was used.
2. After November 2020, a KaVo OP3D Pro (KaVo Kerr, California, USA) with a FOV = 5 cm × 5 cm, and a voxel size of 0.08 mm.

All surgical procedures were performed using a dental operating microscope (Carl Zeiss, Oberkochen, Germany) with a protocol similar to that described in a previous study [13]. The retrograde cavities were filled with either ProRoot MTA (Dentsply Sirona, Charlotte, NC, USA), RRM putty (Brasseler, Savannah, GA, USA), or injected BC sealer (Brasseler USA) followed by the placement of the RRM putty.

### Patient recruitment

All eligible participants were invited to attend a recall appointment. If a patient was unable to attend the recall appointment, the patient’s reason for not attending was recorded.

### Clinical and radiographical evaluation

At the recall appointment, one operator (R.E.) performed the clinical examination, including percussion, palpation, and periodontal examination. For each patient, PA was taken using the paralleling technique, and a limited-FOV CBCT scan (5 cm × 5 cm) with a voxel size of 0.08 mm was taken after written consent was obtained. For the posterior teeth, bitewing radiography was acquired to assess the quality of the coronal restoration. An evaluation form was used to identify the potential prognostic factors of EMS, including patient-related factors (patient’s sex and age), tooth-related factors (tooth type and position, size of periapical radiolucency [PARL, measured on CBCT imaging by determining the widest diameter], and volume of PARL), and treatment-related factors. The treatment-related factors that were investigated in this study were as follows:

1. The length of root canal filling (RCF) was evaluated on PA using the criteria suggested by Sjogren et al. [14]. The filling was considered to be of adequate length if it ended 0–2 mm from the radiographic apex; however, if the length was shorter or longer than this range, the RCF was considered inadequate.
2. The density of RCF was evaluated based on the criteria by Chugal et al. [15]. Adequate density was defined as a homogeneous radiopaque material without voids or space within the body of the filling material or between the material and the walls of the canal. The density of RCF was considered inadequate when the radiopaque material was uniform and/or when the canal space was visible laterally and apically.
3. Preoperative nonsurgical root canal retreatment
4. The angle of root-end resection was measured by the method described by Von Arx et al. [16].
5. The type of root-end filling material was recorded.
6. The depth of the retrograde filling material was measured on CBCT imaging.

### Outcome assessment

Two calibrated board-certified faculty members (M.A. and M.B.) independently assessed all PAs, followed by the CBCT images. After 2 weeks, the examiners independently re-evaluated all the images to determine the intra-observer agreement. During the radiographic evaluation, the examiners were blinded to the clinical diagnosis and type of retrograde filling material used. All radiographic images were observed on a 24-inch HP monitor (EliteDisplay E243, CA, USA) with a resolution of 1920 × 1080 pixels in a dimly lit room without windows.

For the evaluation of PAs, three images were projected for each tooth (preoperative, postoperative, and follow-up), and the tooth was evaluated based on the criteria (complete, incomplete, uncertain, or unsatisfactory healing) established by Rud et al. [17] and Molven et al. [18]. The selected sections of pre- and postoperative CBCT scans were prepared using OnDemand3D software (Cybermed Inc., Daejeon, South Korea) by an endodontist (R.E.), who was not involved in the radiographic assessment. The selected sections were arranged in a cross-sectional view by placing the center of the vertical and horizontal reference lines in the axial view through the center of the root and then aligning the vertical reference lines in mesiodistal and buccolingual views through the center of the root along its longitudinal axis [19].

**Fig. 1:**
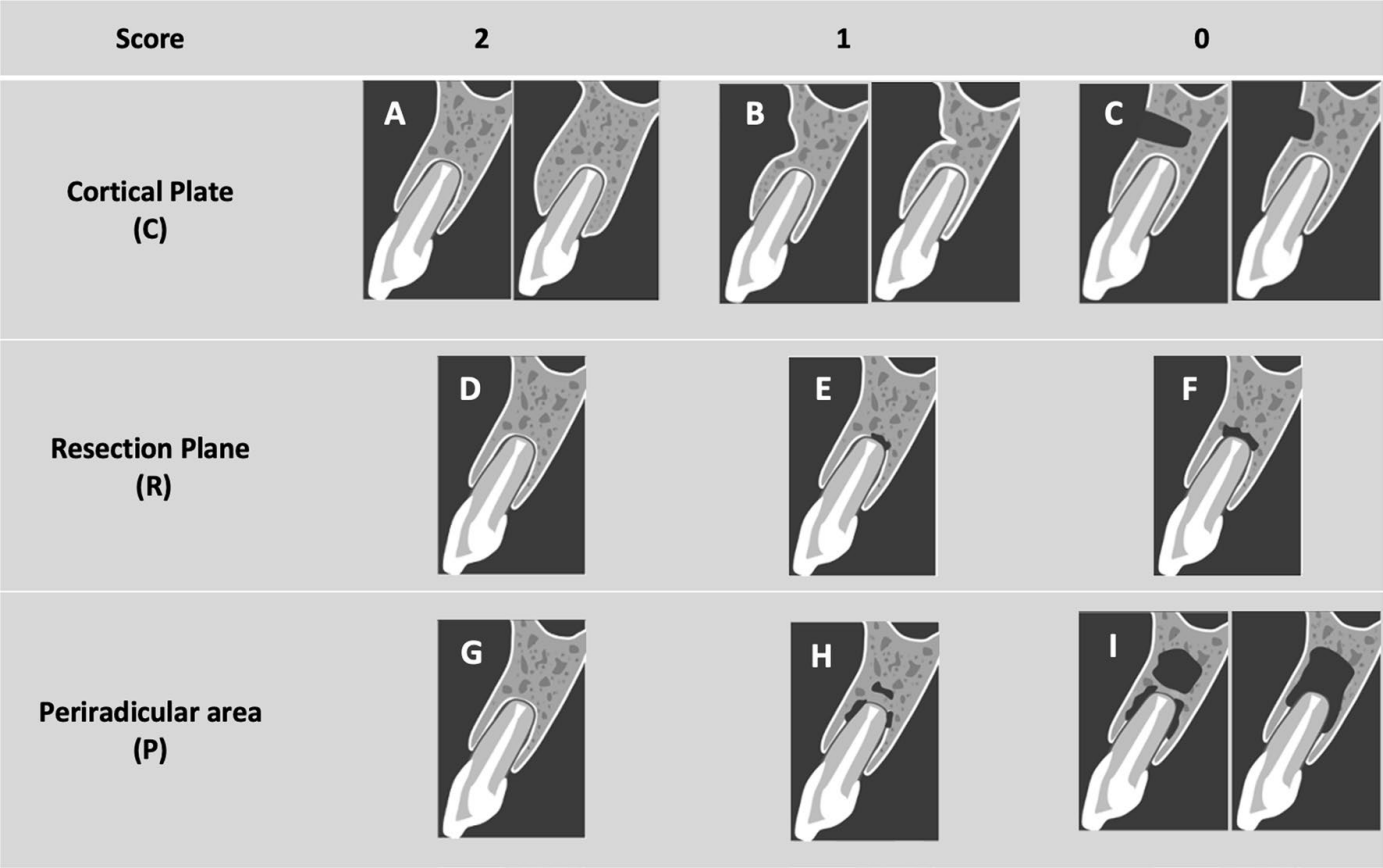
The modified Chen et al. criteria for 3-dimensional radiographic evaluation after endodontic microsurgery. Cortical plate (access bony window) C-index. (**A**) Score 2: the cortical plate is re-established and flat. (**B**) Score 1: the cortical plate is re-established, but concave or with minor indentations. (**C**) Score 0: the cortical plate has not been re-established or is only partially re-established. Resection plane (cut root face) R-index. (**D**) Score 2: complete bone deposition on the resection plane (complete formation of periodontal ligament [PDL] space of normal width). (**E**) Score 1: partial bone deposition on the resection plane (partial formation of PDL space). (**F**) Score 0: no bone deposition on the resection plane (no formation of PDL). (**G**) Periradicular area P-index. (**G**) Score 2: complete bone formation in the periradicular area. (**H**) Score 1: partial bone formation in the periradicular area that appears to be smaller than the lesion size in the preoperative cone-beam computed tomography regardless of the R or C indices. (**I**) Score 0: no apparent bone formation around the periradicular area regardless of the R or C index

The modified Chen et al. criteria by Azim et al. [8, 10] were used to evaluate the preselected pre- and postoperative CBCT images of each tooth to assess 3D healing (Fig. 1). These criteria comprised three indices: C (cortical plate), R (resection plane), and P (periradicular area). Each tooth received a score of 0, 1, or 2 for each index, after which the scores of the three indices were summed (C + R + P) to obtain a final score. The final score per tooth ranged between 0 and 6, and scores were classified the following 3D radiographic healing category established by Azim et al. [10]:

1. Complete healing: final score = 6
2. Incomplete healing: score = 5 or 4
3. Uncertain healing score = 3
4. Unsatisfactory healing: score = 2, 1, or 0

For multi-rooted teeth, the outcome was determined based on the root showing the worst prognosis. In case of disagreement regarding the radiographic healing category, the examiners re-discussed the case until a consensus was reached. The outcome was categorized as success or failure based on the clinical and radiographic evaluation, as follows:

1. Success, if the case was categorized as complete or incomplete radiographic healing without clinical signs and/or symptoms.
2. Failure, if the case was categorized as uncertain or unsatisfactory radiographic healing with or without clinical signs and symptoms. Cases with signs and/or symptoms during the recall appointment were considered a failure regardless of the evidence of radiographic healing.

### Volumetric measurement of PARLs

Volumetric analysis of PARLs on both preoperative and follow-up CBCT scans was performed using Amira software (version 2021.1; Visage Imaging, Berlin, Germany) following a protocol similar to that described in a previous study [11]. The borders of the PARL were determined by consensus between an endodontist and a radiologist. Multiple PARLs per tooth or combined PARLs were evaluated as a single volume.

### Statistical analysis

The chi-square and Fisher’s exact tests were used to identify the significant prognostic factors with the outcome (clinical & CBCT), and subsequently with CBCT healing categories. Binary logistic regression was performed to assess the predictability of the follow-up time on the outcome of EMS. Cohen’s kappa test was used to assess inter- and intra-observer agreements, as well as to assess the agreement between PA and CBCT imaging in evaluating the outcome of EMS. A paired t-test was performed to measure the mean difference between the pre- and postoperative PARLs volumes. All statistical analyses were performed as 2-tailed tests with the level of significance set at *P* < *0.05* using SPSS v25.0 software (IBM Corp, Armonk, NY).

## Results

**Fig. 2:**
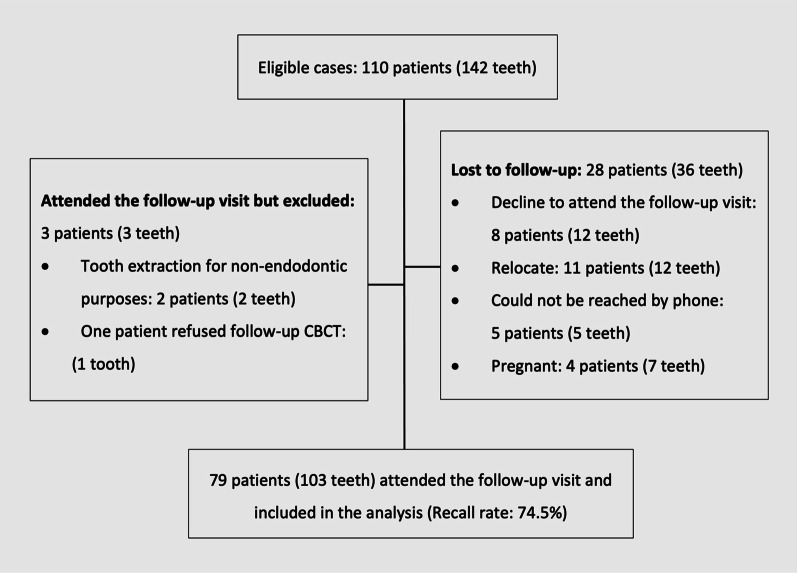
Flow chart of participation eligibility and recall rate

Among the 110 patients (142 teeth) eligible for follow-up, 79 patients (103 teeth/127 roots) attended the follow-up visits and were included in the analysis. Twenty-eight patients were lost to follow-up (recall rate = 74.5%) (Fig. 2). The study population consisted of 30 males and 49 females with a mean age of 36 years (age range = 16–61 years). Of the 103 teeth, 73 teeth were in the maxilla and 30 were in the mandible; in total, 40 anteriors, 24 premolars, and 39 molars were included. The follow-up period ranged from 12 to 55 months, with an average of 24.36 months.

The combined success rate of EMS as evaluated on PAs was 88.4%, with a success rates of 85.7% for MTA, 85.4% for RRM putty, and 94.1% for the lid technique. No statistically significant difference was noted among the three groups. The overall success rate of EMS as evaluated on CBCT images was 77.7%, with a success rates of 67.9% for MTA, 75.6% for RRM putty, and 88.2% for the lid technique; these group-specific rates were not significantly different (Table 1).

**Table 1:** Association between retrograde filling materials and the outcomes of endodontic microsurgery evaluated by two imaging modalities

	Clinical + PA			Clinical + CBCT imaging			
	Success	Failure		Success		Failure	
	Teeth	n (%)	n (%)	*P* value	n (%)	n (%)	*P* value
MTA	28	24 (85.70)	4 (14.30)	0.440	19 (67.90)	9 (32.10)	0.146
RRM putty	41	35 (85.40)	6 (14.60)		31 (75.60)	10 (24.40)	
Lid technique	34	32 (94.10)	2 (5.90)		30 (88.20)	4 (11.80)	

*PA* periapical radiography, *CBCT* cone-beam computed tomography, *MTA* mineral trioxide aggregate, *RRM* putty, root repair material putty

**Fig. 3:**
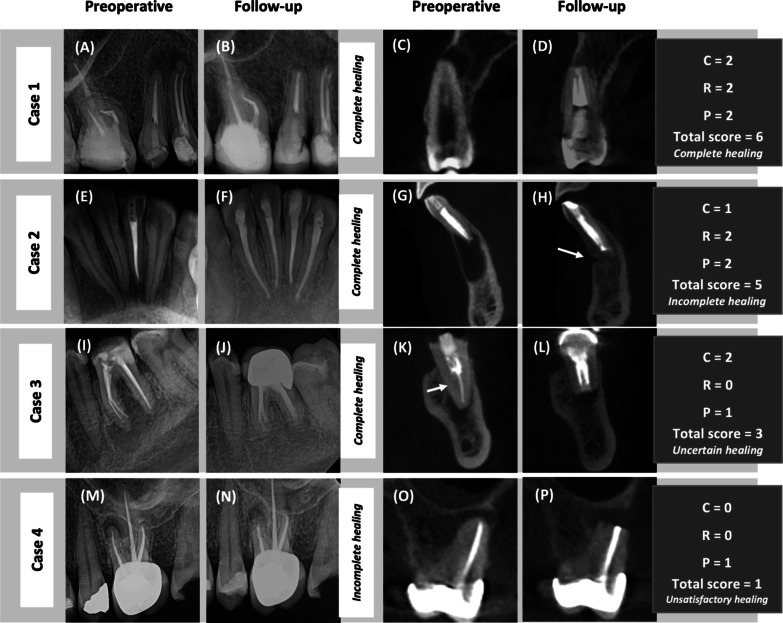
Preoperative and follow-up periapical radiographs (PAs) and cone-beam computed tomography (CBCT) images in corrected buccolingual views showing different patterns of radiographic healing. Case 1: (**A**–**D**): Maxillary second premolar presented at two-years after the surgery. (**A** and **B**) PAs showed complete healing. (**C** and **D**) Example of complete healing with respect to all three indices. Case 2: (**E**–**H**): Mandibular left central incisor that was followed up after one-year and eight-months. (**E** and **F**) Example of complete healing in PA. (**G** and **H**) Example of incomplete healing in CBCT. The arrow points to the cortical plate (C-index), which showed minor indentation and received a score of 1; meanwhile, the resection plane (R) and periradicular area (P) showed complete bone deposition, receiving a score of 2 each. Case 3: (**I**–**L**): Mandibular first molar at the one-year follow-up. (I) PA showing periapical radiolucency (PARL) associated with both mesial (M) and distal (D) roots. (**J**) Follow-up PA showed complete healing, but the density of the bone on the surgical side of the D root was lower than of the surrounding bone. (**K**) Preoperative CBCT showed PARL related to the D root of the first molar. The arrow points to the missed distolingual canal. (**L**) Follow-up CBCT showed complete re-establishment of the C-index (score 2) and partial improvement in the P-index (score 1), whereas no improvement was observed in the R-index (score 0). Case 4: (**M**–**P**) Maxillary first molar presented at one-year and six-months after the surgery with clinical signs and symptoms of failure. (**M** and **N**) PAs showing incomplete healing. (**O**) Preoperative CBCT of the mesiobuccal root with PARL. (**P**) Follow-up CBCT: the P-index reflects partial bone formation (a score of 1), but C- and R- indices reflect a lack of bone deposition (a score of 0 each)

Twelve cases were clinically symptomatic to percussion and were considered failures. In the 2D analysis, only one case was classified as uncertain healing and considered a failure. In the 3D analysis, 18 cases were considered failures (nine cases with uncertain healing and nine cases with unsatisfactory healing) (Table 2). Figure 3 shows four cases with different healing categories on CBCT imaging, while PA shows only two categories, either complete or incomplete healing.

**Table 2:** Distribution of cases according to 2-dimensional (2D) and 3-dimensional (3D) healing categories

Healing category	Complete	Incomplete	Uncertain	Unsatisfactory
	n (%)	n (%)	n (%)	n (%)
2D	94 (91.26)	8 (7.77)	1 (0.97)	0 (0.00)
3D	50 (48.53)	35 (33.98)	9 (8.74)	9 (8.74)

Overall, there was a slight agreement observed between the PA and CBCT outcomes with a statistically significant difference (Cohen’s kappa (K) = 0.088; *P* = 0.029). The Cohen’s Kappa value of 2D analysis revealed substantial intra and inter-observer agreements ((K = 0.750; *P* < 0.001) (K = 0.647; *P* < 0.001), respectively). The Cohen’s Kappa value of 3D analysis showed moderate intra-observer (K = 0.572; *P* < 0.001) agreement and substantial inter-observer agreement (k = 0.677; *P* < 0.001), in accordance with the Landis and Koch criteria [20].

None of the patient-, tooth-, or treatment-related factors significantly influenced the binary outcome of EMS (success/failure) (Table 3). Adequate density of RCF was significantly associated with a high percentage of completely healed cases (58.9%) as assessed using CBCT imaging (*P* = 0.044). A binary logistic regression model revealed no significant relationship between the follow-up time and EMS outcomes (*P* = 0.689).

**Table 3:** Distribution of the variables and analysis of the prognostic factors

	Outcome (Clinical + CBCT)			CBCT healing category				
	Tooth/ Root	Success		Complete	Incomplete	Uncertain	Unsatisfactory	
Factor	n (%)	n (%)	*P* value	n (%)	n (%)	n (%)	n (%)	*P* value
*Sex*								
Male	37 (35.90)	26 (70.27)	0.220	21 (56.76)	8 (21.62)	5 (13.51)	3 (8.11)	0.157
Female	66 (64.10)	54 (81.82)		29 (43.94)	27 (40.91)	4 (6.06)	6 (9.09)	
*Age*								
≤ 45 year	81 (78.60)	62 (76.54)	0.775	38 (46.91)	29 (35.80)	7 (8.64)	7 (8.64)	0.886
> 45 year	22 (21.40)	18 (81.82)		12 (54.55)	6 (27.27)	2 (9.09)	2 (9.09)	
*Tooth type*								
Anterior	40 (38.83)	29 (72.50)	0.428	18 (45.00)	14 (35.00)	5 (12.50)	3 (7.50)	0.738
Premolar	24 (23.30)	21(87.50)		15 (62.50)	7 (29.17)	1 (4.17)	1 (4.17)	
Molar	39 (37.86)	30 (76.92)		17 (43.59)	14 (35.90)	3 (7.69)	5 (12.82)	
*Tooth position*								
Maxillary	73 (70.90)	58 (79.45)	0.603	38 (52.05)	24 (32.88)	6 (8.22)	5 (6.85)	0.549
Mandibular	30 (29.10)	22 (73.33)		12 (40.00)	11 (36.67)	3 (10.00)	4 (13.33)	
Size of PARL*								
≤ 5 mm	84 (66.10)	65 (77.4)	0.828	50 (59.52)	24 (28.57)	4 (4.76)	6 (7.14)	0.146
> 5 mm	43 (33.90)	34 (79.07)		18 (41.86)	16 (37.21)	6 (13.95)	3 (6.97)	
*Volume of PARL*								
≤ 70 mm^3^	50 (50.50)	37 (74.00)	0.361	26 (52.00)	15 (30.00)	2 (4.00)	7 (14.00)	0.150
> 70 mm^3^	49 (49.50)	40 (81.63)		22 (44.90)	19 (38.78)	6 (12.24)	2 (4.08)	
*Length of RCF*								
Adequate	59 (57.30)	47 (79.66)	0.574	30 (50.85)	21 (35.59)	3 (5.08)	5 (8.47)	0.508
Inadequate	44 (42.70)	33 (75.00)		20 (45.45)	14 (31.82)	6 (13.64)	4 (9.09)	
*Density of RCF*								
Adequate	56 (54.40)	47 (83.93)	0.096	33 (58.93)	18 (32.14)	2 (3.57)	3 (5.36)	0.044**
Inadequate	47 (45.60)	33 (70.21)		17 (36.17)	17 (36.17)	7 (14.89)	6 (12.77)	
*Preoperative nonsurgical retreatment*								
Yes	30 (29.10)	26 (86.66)	0.199	13 (43.33)	14 (46.67)	2 (6.67)	1 (3.33)	0.335
No	73 (70.90)	54 (73.97)		37 (50.68)	21 (28.77)	7 (9.59)	8 (10.96)	
Angle of root-end resection*								
≤ 20°	114 (89.80)	88 (77.19)	0.732	61 (53.51)	35 (30.70)	9 (7.89)	9 (7.89)	0.854
> 20°	13 (10.20)	11 (84.62)		7 (53.85)	5 (38.46)	1 (7.69)	0 (0.0)	
Depth of retrograde filling material*								
< 2.5 mm	97 (76.40)	73 (75.26)	0.218	50 (51.55)	29 (29.89)	10 (10.31)	8 (8.25)	0.234
≥ 2.5 mm	30 (23.60)	26 (86.67)		18 (60.00)	11 (36.67)	0 (0.00)	1 (3.33)	

*Variables assessed per root

**Indicates significance; *PARL* periapical radiolucency; *RCF* root canal filling

Four teeth were excluded from volumetric analysis because their preoperative CBCT scans could not be imported into Amira software. The preoperative volume of PARLs was reduced in 88.9% of the cases (n = 88), remained unchanged in 1.0% (n = 1), and increased in 10.1% of the cases (n = 10) at follow-up. There was a significant difference between the mean preoperative PARL volumes (mean = 238.04 mm^3^, n = 99, standard deviation [SD] 473.83 mm^3^) and the mean postoperative PARL volumes (mean = 21.74 mm^3^, n = 99, SD 31.429 mm^3^) (*P* < 0.001). The mean volume reduction was 57.5%.

## Discussion

This retrospective study aimed to evaluate the outcome of first-time EMS that involved retrograde filling with MTA, RRM putty or, the lid technique by using two imaging modalities. The outcome of EMS combined with GTR has been reported as a factor significantly associated with favorable healing using CBCT imaging [10]. Therefore, GTR and bone grafts are considered external factors that may mask radiographic bone healing. Hence, patients who underwent GTR/bone grafting were excluded from this study.

Three-dimensional healing assessment was performed according to the modified Chen et al. criteria [8, 10]; however, the interpretation of a C-index score of 0 was modified to adapt to our exclusion criteria of cases with through-and-through defect, and specified into either no or partial re-establishment of the buccal cortical plate.

### Retrograde filling materials

The lid technique follows the concept of using two materials, similar in chemical composition but different in consistency, to achieve maximum adaptation to the biological surfaces [21]. In an in vitro study, Rencher et al. [22] compared the sealing ability of the lid technique, MTA, and BC putty as retrograde filling material. Scanning electron microscopy showed adequate adaptation of all the materials to the dentinal walls, with no significant difference in the sealing ability between the groups. In the present study, the success rate of the lid technique was high and comparable to the success rates reported previously (92.0% on PA, and 93.3% on CBCT imaging) [10, 23]. However, the success rates of MTA and RRM putty on PA and CBCT imaging were lower than the previously reported success rates (92–94% on PA and 85–92% on CBCT imaging) [9, 10]. This difference may be attributed to the small sample size and uneven distribution of cases per group. Nevertheless, our results revealed no statistically significant differences in the 2D or 3D outcomes among the three groups of retrograde filling materials, which is in agreement with the Buffalo study [10].

### Imaging modalities

Our results showed that the success rate of EMS according to PA was 10.7% higher than the rate according to CBCT imaging. This result corresponded to the reported 3–8% difference between the success rates of the two imaging modalities in recent studies [9, 10]. Previous studies have found that CBCT is more accurate than PA in determining bone healing [11, 12]. Schloss et al. [12] found a significant difference between 2 and 3D healing classifications. CBCT was more precise in evaluating incomplete and uncertain healing statuses than PA. These results were consistent with our findings. CBCT imaging was able to detect nine cases each of uncertain and unsatisfactory healing, whereas PA detected only one case of uncertain healing. This difference in outcome assessment between the two imaging modalities may have originated from the ability of CBCT imaging to visualize healing in the buccolingual dimension and cortical bone formation, which could not be observed on PA. This indicated that CBCT imaging is more precise than PA in detecting uncertain and unsatisfactory healing.

### Prognostic factors

The effect of potential prognostic factors was assessed first on combined clinical and CBCT outcomes and then on CBCT healing categories. None of the factors significantly affected the outcomes. These results were consistent with those of previous reports that examined the sex, tooth type and position, preoperative nonsurgical retreatment [9, 10, 23], patient age and size of PARL [23], length of RCF [9], and angle of root-end resection [16].

The depth of retrograde fillings was not found to be a significant factor, which was in accordance with the finding of the Buffalo study [10]. Nevertheless, this finding was not in concordance with that of the study by Safi et al.[9], in which it was reported that inadequate depth of filling (< 2.5 mm) was significantly associated with EMS failure. The lack of significance in our study may be related to the uneven distribution of cases among the groups. Further studies including deeper retrograde cavities are required to compare the effect of the three retrograde filling groups (MTA, BC putty, and lid technique) on EMS outcomes.

In the meta-analysis by Von Arx et al. [24], cases with an adequate density of RCF showed a significantly increased success rate. However, in our study, an adequate density of RCF did not significantly affect the outcome, whereas it was significantly associated with a high percentage of completely healed cases on CBCT during the follow-up period of 1–4 years. This result was comparable to the findings of Safi et al. [9], who reported that inadequate density of RCF was significantly associated with failure cases on CBCT imaging. This suggested that an adequate density of RCF with well-condensed gutta-percha minimizes the bacterial reservoir in the root canal system, thereby providing an adequate environment and proper seal to achieve complete healing [9].

In a previous study by Kim et al. [25], the median PARL volume (50 mm^3^) was used as the cut-off point to create two groups for comparison. They reported that cases with preoperative PARL volumes above 50 mm^3^ were significantly associated with failure. In the present study, the median PARL volume was 70 mm^3^, and PARL volumes above this value did not significantly affect the outcome. This difference in the results may be attributed to the difference in the inclusion criteria, the method of volume measurement, and the software used.

### Volumetric changes

PARL volumes reduced significantly over the follow-up period (1–4 years); this was observed in 88.9% of cases. A similar significant reduction was also detected in a 1-year follow-up study by Kang et al. [26], and in the study by Schloss et al. [12], which had a 1- to 3-year follow-up period. In addition, the percentage of reduction in our study was comparable to that reported by Curtis et al. [11] (97.8%). However, an increase in PARL volume was observed in 10 cases in our study. Six cases with increased volume were classified as unsatisfactory healing on 3D analysis, and four cases showed evidence of excessive bone removal during the surgery.

CBCT had superior ability than PA in detecting PA lesion after endodontic treatment, because CBCT overcome the limitations of 2D radiographs, which include anatomical overlap and geometric distortion [27]. A systematic review by Antony et al. [28] concluded that CBCT had higher accuracy than panoramic radiograph and PA in detecting PA lesions after endodontic treatment. Regarding concerns about radiation exposure, the patients in our study underwent follow-up CBCT in the limited-FOV mode at least 1 year after the surgery, which has been found to be an adequate time to assess healing after EMS [29]. This follow-up protocol maximizes the benefits for the patient.

This study targeted a specific group of patients (first-time EMS recipients with no bone graft or GTR), which enabled us to observe the pure effect of the prognostic factors on the outcome. Moreover, the radiographic evaluation used a 3D imaging modality that allowed an accurate outcome assessment. Nevertheless, this retrospective study was limited by the inclusion of more than one tooth per patient. In addition, the data were retrieved from a single center (KAUDH); therefore, there was a lack of external validation. Furthermore, there was heterogeneity in the follow-up time between the cases. However, binary logistic regression revealed that the follow-up time did not significantly predict the outcome. Our results present valuable initial data that can be used as a baseline for future multi-center prospective cohort studies or randomized controlled trials with larger sample sizes.

## Conclusion

EMS is a predictable procedure with high success rates observed on both 2D and 3D imaging, when MTA, RRM putty, or the lid technique as retrograde filling materials. The adequate density of RCF is a significant factor associated with complete healing on CBCT. A significant difference was noted between PA and CBCT outcomes. CBCT was able to detect failed cases more efficiently than PA. PARL volumes were reduced significantly over 1–4 years follow-up after EMS. CBCT is a valuable tool to assess the volumetric changes after EMS.

## Data Availability

The datasets used and analyzed during the current study are available from the corresponding author on reasonable request.

## Abbreviations

EMS: Endodontic microsurgery
MTA: Mineral trioxide aggregate
RRM: Root repair material
BC: Bioceramic
PAs: Periapical radiographs
CBCT: Cone-beam computed tomography
PARLs: Periapical radiolucencies
ES: Endodontic surgery
GTR: Guided tissue regeneration
2D: 2-Dimensional
3D: 3-Dimensional
ASA: American society of anesthesiologists
FOV: Field of view
RCF: Root canal filling
PDL: Periodontal ligament
